# Taxonomic richness and abundance of cryptic peracarid crustaceans in the Puerto Morelos Reef National Park, Mexico

**DOI:** 10.7717/peerj.3411

**Published:** 2017-06-15

**Authors:** Luz Veronica Monroy-Velázquez, Rosa Elisa Rodríguez-Martínez, Fernando Alvarez

**Affiliations:** 1 Colección Nacional de Crustáceos, Instituto de Biología, Universidad Nacional Autónoma de México, Ciudad de México, México; 2 Instituto de Ciencias del Mar y Limnología, Universidad Nacional Autónoma de México, Puerto Morelos, Quintana Roo, México

**Keywords:** Coral rubble, Mexican Caribbean sea, Coral reef, Peracarida, Cryptic crustaceans

## Abstract

**Background and Aims:**

Cryptic peracarids are an important component of the coral reef fauna in terms of diversity and abundance, yet they have been poorly studied. The aim of this study was to evaluate the taxonomic richness and abundance of cryptic peracarids in coral rubble in the Puerto Morelos Reef National Park, Mexico (PMRNP), and their relationship with depth.

**Methods:**

Three reef sites were selected: (1) Bonanza, (2) Bocana, and (3) Jardines. At each site six kilograms of coral rubble were collected over four sampling periods at three depths: 3 m (back-reef), 6–8 m (fore-reef), and 10–12 m (fore-reef).

**Results:**

A total of 8,887 peracarid crustaceans belonging to 200 taxa distributed over five orders and 63 families was obtained; 70% of the taxa were identified to species and 25% to genus level. Fifty species of those collected represent new records for the Mexican Caribbean Sea. Isopoda was the most speciose order while Tanaidacea was the most abundant.

**Discussion:**

Cryptic peracarid taxonomic richness and abundance were related to depth with higher values of both parameters being found in the shallow (3 m) back-reef, possibly due to a higher reef development and a greater accumulation of coral rubble produced during hurricanes. Peracarid data obtained in the present study can be used as a baseline for future monitoring programs in the PMRNP.

## Introduction

Coral reefs are one of the most complex and productive ecosystems of the world and support one of the highest diversity of the marine realm due to its highly complex architecture (Glynn & Enochs, 2011). Both live and dead coral provide essential habitat and shelter for symbiotic and cryptic species, including polychaetes, gastropod mollusks, echinoderms and crustaceans. These species inhabit cracks or holes, formed by bioeroders, or in the interstices between coral rubble and dead corals, or that nestle within reef framework (Takada, Abe & Shibuno, 2007; Enochs et al., 2011). Among crustaceans, peracarids are dominant taxonomic components of the reef cryptofauna and play an important ecological role within the reef ecosystem as they have a position near the base of various food chains, consume epiphytic algae, and recycle organic matter and detritus (Kensley, 1984; Preston & Doherty, 1990; Hernández et al., 2014). Despite the high diversity and abundance of reef cryptic fauna, it has been seldom studied (Enochs et al., 2011), largely due to difficulties in collecting and identifying species (Enochs, 2010; Plaisance et al., 2011).

Coral reefs have deteriorated in the last decades world-wide because of climate change, diseases, macroalgal overgrowth, overfishing, sedimentation, low water quality and hurricanes (Diaz-Pulido et al., 2009; Sotka & Hay, 2009). Decline in coral coverage has resulted in shifts from coral-dominated to macroalgae-dominated reefs (Hughes, 1994) and in an accelerated loss of architectural complexity (Álvarez-Filip et al., 2011). Given this decline there is a pressing need to understand how cryptofauna is organized and how it may respond to further declines in environmental parameters. Coral reef cryptofauna can also be used as a bioindicator of environmental degradation due to changes in abundance, presence/absence, condition and behavior (Linton & Warner, 2003; Takada, Abe & Shibuno, 2008). Among cryptofauna, peracarids are excellent candidates for ecological studies because they lack a pelagic larval state, have specific habitat requirements, and exhibit low intrinsic rates of dispersal (Thomas, 1993a). Amphipoda, for example, have been found to be more sensitive than other groups of invertebrates (i.e., decapods, polychaetes, molluscs, and asteroids) to a variety of contaminants (Ahsanullah, 1976; Swartz et al., 1985; Swartz, 1987) and to show responses to dredging, shoreline alteration, fishing practices and salinity (Barnard, 1958; Barnard, 1961; McCluskey, 1967; McCluskey, 1970). The usefulness of amphipods as bioindicators has been recognized by some government agencies, which now require their identification to the species level in permitting operations such as oil leases (Linton & Warner, 2003). However, their incorporation into bioassessment programs on coral reefs is dependent upon completion of comprehensive coastal resource inventories and taxonomic surveys (Thomas, 1993a).

The objective of this study was to make a quantitative assessment of the taxonomic richness and assemblage composition of peracarids in coral rubble within the Puerto Morelos Reef National Park, Mexico. We also address two research questions: (i) Do taxonomic richness and abundance of cryptic peracarids vary with depth?; and (ii) do these parameters vary between reef sites?

## Materials and Methods

### Study sites

**Figure 1:**
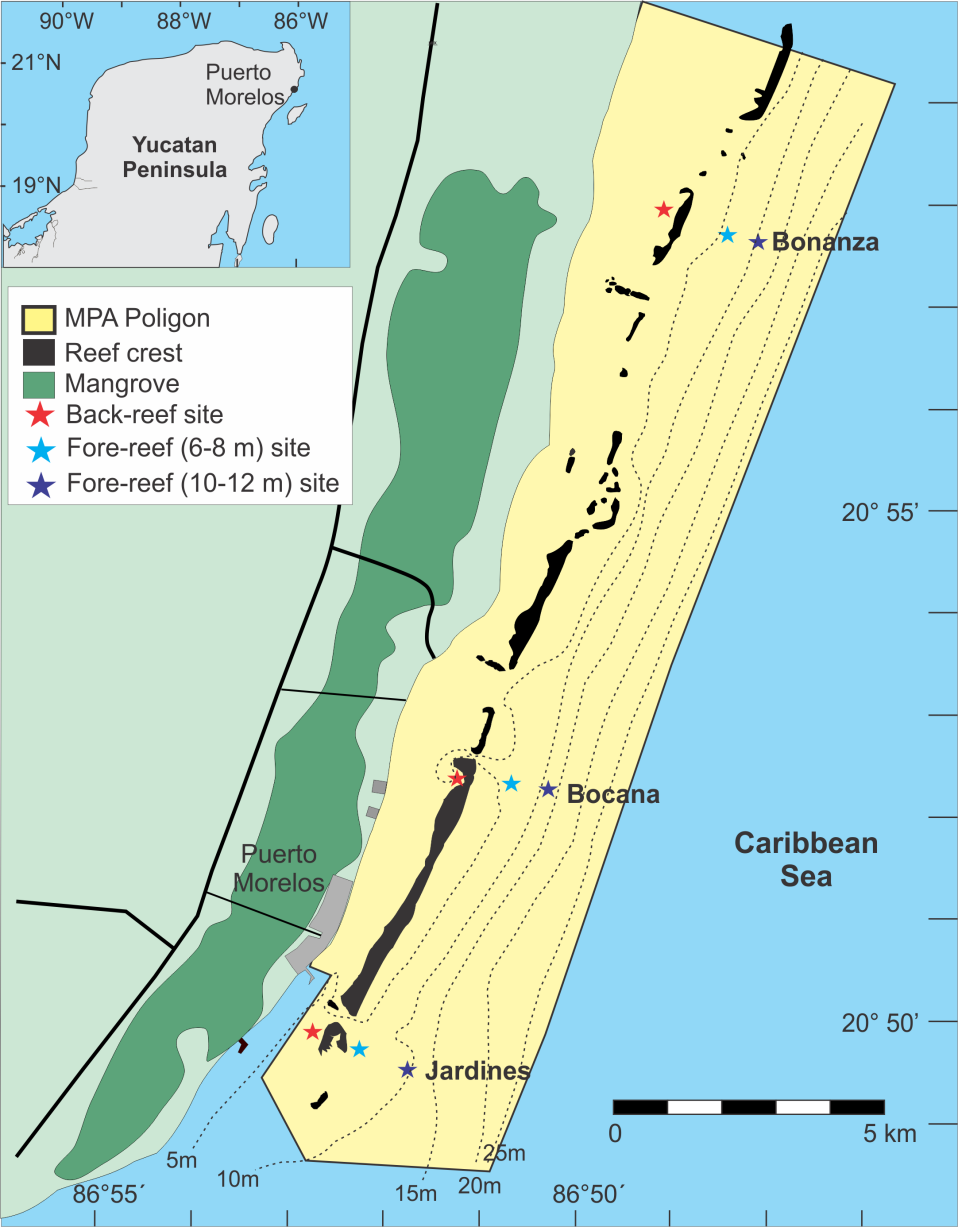
Location of the study sites (figure modified from Monroy-Velázquez & Alvarez (2016)). The color of the star indicates the reef depth where the samples were collected.

The study site is located within the Puerto Morelos Reef National Park (PMRNP), in Quintana Roo, Mexico (Fig. 1). This marine protected area (MPA) was created in 1998, and has an area of 9,066 ha, extending for 21 km along the NE coast of the Yucatan Peninsula and from the beach to 4.5–5 km seaward (Fig. 1). The MPA contains a fringing reef that is close to shore (<3.5 km) which has been described in several papers (Jordán, 1979; Ruiz-Rentería, Tussenbroek & Jordán-Dahlgren, 1999; Rodríguez-Martínez et al., 2010). Details on the creation of the MPA, its management, and the major problems it faces have also been described (Rodríguez-Martínez, 2008). At present, major threats to the PMRNP are climate change and tourism related urban development (Hernández-Terrones et al., 2011; Rodríguez-Martínez et al., 2010).

Three reef sites were selected: (1) Bonanza (20°57′58″N, 086°48′27″W), (2) Bocana (20°52′50″N, 086°51′02″W), and (3) Jardines (20°50′20″N, 086°52′41″W) (Fig. 1). The distance between sites was approximately 10 km. Tourist activities are conducted in the back-reef zone of all three reef sites and in the reef front of Jardines, with snorkeling being the dominant activity in Bonanza and Bocana and SCUBA diving in Jardines. Fishing is only allowed in the fore-reef of Bonanza, however, since the MPA is narrow (≤5 km) fishing at its edges could have an effect on the other two surveyed sites.

### Sampling

In order to quantitatively sample cryptic peracarids, six kilograms of coral rubble were collected randomly at each reef site from three depths: 3 m (back-reef), 6–8 m (fore-reef), and 10–12 m (fore-reef); hammer and chisel were used when the coral rubble was consolidated. One sample was collected by SCUBA divers from each depth, at each site, in four months (May, August, and November 2013, and January 2014). Samples were placed in plastic bags *in situ* and immediately transported to the laboratory, where fragments were placed in buckets with fresh water to induce osmotic shock and force cryptofauna to leave the microhabitats (holes and crevices) (Ochoa-Rivera, Granados-Barba & Solis-Weiss, 2000). Consolidated coral rubble was broken into smaller pieces with chisel to extract all organisms; the remainder of the sample was sieved through a 0.5 mm mesh. Organisms were fixed in 70% ethanol for later sorting and identification. Identification keys used were those of Thomas (1993b), LeCroy (2000), LeCroy (2002), LeCroy (2004) and LeCroy (2007) for Amphipoda, Kensley & Schotte (1989) for Isopoda, Suárez-Morales et al. (2004) for Tanaidacea, and Heard, Roccatagliata & Petrescu (2007) for Cumacea. When identification to species level was not possible, and the specimen was clearly a different taxon from others collected, a letter was used to characterize the species (i.e., species A); this allowed these taxa to be taken in account for the calculation of taxonomic richness. All surveys were conducted under permit DGOPA.00008.080113.0006 granted by SAGARPA (Agriculture, Natural Resources and Fisheries Secretariat) to F Alvarez.

### Data analysis

Similarities in peracarid taxonomic richness among reef sites and depths were summarized in Venn diagrams. The hypothesis that cryptic peracarid abundance varied with (1) reef site and (2) depth was tested using a 2-factor ANOVA, using the four sampling surveys as replicates. Abundance data were transformed by log10(x) prior to the statistical analysis. Homogeneity of variances was confirmed by Bartlett’s test (*p* > 0.05). To visualize differences in the dominant taxa across sites and depths we constructed a heatmap (a visualization technique where cells in a matrix with high relative values are colored differently from those with low relative values) and a hierarchical clustering, performed with the average linkage method from a Bray–Curtis dissimilarity matrix; all taxa were used to do the hierarchical clustering but only the more abundant taxa are displayed in the heatmap (those whose relative abundance was higher than 5%). All analyses were done in R (R Core Team, 2016) using packages: ggplot2 (Wickham, 2009), plyr (Wickham, 2014), gplots (Warnes et al., 2009), vegan (Oksanen et al., 2017) and RColorBrewer (Neuwirth, 2011). A reproducible record of all statistical analyses is available on GitHub (https://github.com/rerodriguezmtz/Peracarids). This includes all underlying data and R code for all analyses.

## Results

A total of 8,887 specimens of cryptic peracarids were collected from coral rubble consisting of 200 taxa, and belonging to five orders and 63 families; 141 taxa were identified to species-level, 50 only to generic-level and nine only to family-level (Table 1). Among these, Isopoda was the most speciose order, with 75 taxa and 2,346 individuals, followed by Amphipoda, with 72 taxa and 1,416 individuals, and then Tanaidacea had the largest abundance, with 22 taxa and 4,942 individuals; Cumacea was represented by 30 taxa, but accounted for only 2% of the collected organisms, and Mysidacea was represented by a single family and only three specimens. Among isopods, 473 of the individuals were larvae and females from the *Gnathia* genera that could not be identified to species level, as identification keys are based solely on the morphology of adult males (Kensley & Schotte, 1989). The most speciose families were Anthuridae (Isopoda, with 16 taxa), Nannastacidae (Cumacea, with 13 taxa) and Maeridae (Amphipoda, with 12 taxa). Fifty species (25%) of those collected represent new records for the Mexican Caribbean Sea (in bold letters in Table 1); 19 of them were previously reported by Monroy-Velázquez & Alvarez (2016).

**Table 1:** Number of individuals (*N*) of five Peracarid crustacean’s taxa collected in coral rubble from three reef sites (Bz: Bonanza, Bo: Bocana, Ja: Jardines) and three depths (S: shallow back-reef, M: medium depth fore-reef (6–8 m), D: deep fore-reef (10–12 m)) within the Puerto Morelos Reef National Park in 2013–2014. Bold letters indicate new records for the Mexican Caribbean.

Order	Family	Taxon	*N*	Reef site	Depth
Mysida	Mysidae	Mysidae A	3	Ja	M
Amphipoda	Phliantidae	*Paraphinotus seclusus*	4	Bz, Jar	S, M, D
	Aoridae	*Bemlos spinicarpus*	1	Jar	M
		*Bemlos unicornis*	1	Bo	M
		*Bemlos* sp A	3	Bz	S, D
		*Globosolembos smithi*	62	Bz, Bo, Ja	S, M, D
		*Lembos unifasciatus*	1	Bo	M
	Gammaridae	*Gammarus mucronatus*	2	Bo	S
	Hyalidae	*Apohyale* sp A	1	Bo	S
		Hyalidae A	1	Ja	M
	Chevaliidae	*Chevalia aviculae*	461	Bz, Bo, Ja	S, M, D
		*Chevalia* sp A	2	Bz, Bo	D
	Photidae	*Gammaropsis atlantica*	67	Bz, Bo, Ja	S, M, D
		*Photis* sp A	3	Bo	S
	Biancolonidae	*Biancolina* sp A	1	Bo	S
	Ampithoidae	*Ampithoe ramondi*	30	Bz, Bo, Ja	S, M, D
		*Ampithoe* sp A	14	Bz, Bo, Ja	S, M, D
		*Cymadusa* sp A	12	Bz, Bo, Ja	S, M, D
		*Pseudoampithoides incurvaria*	47	Bz, Bo, Ja	S, M, D
	Caprellidae	*Deutella incerta*	1	Bz	S
		*Hemiproto wigleyi*	8	Bz, Bo	M, D
	Isaeidae	*Caribboecetes* sp A	3	Bo, Ja	S, M
		*Erichthonius brasiliensis*	8	Bo, Ja	S, M
	Eriopisidae	*Psammogammarus* sp A	7	Bz, Bo, Ja	S, M
	Maeridae	*Anamaera hixoni*	1	Ja	S
		*Maera jerrica*	7	Bz, Ja	S, M
		*Maera miranda*	5	Ja	S
		*Maera* sp A	1	Bo, Ja	M
		*Maeropsis* sp A	1	Bo	S
		*Quadrimaera* sp A	12	Bo, Ja	S, M
		*Ceradocus sheardi*	63	Bz, Bo, Ja	S, M, D
		*Ceradocus shoemakeri*	4	Bz	D
		*Dumosus* sp A	12	Bo, Ja	S, M
		*Elasmopus balkomanus*	2	Ja	S
		*Elasmopus levis*	24	Bz, Ja	S, M, D
		*Elasmopus rapax*	190	Bz, Bo, Ja	S, M, D
	Melitidae	*Melita sheardi*	1	Ja	M
		*Melita* sp A	11	Bz, Ja	S, M, D
		*Netamelita barnardi*	9	Jar	S
		***Spathiopus looensis***	7	Bo, Ja	S, M
		*Tabatzius muelleri*	13	Bz, Bo	S, M, D
	Ampeliscidae	*Ampelisca abdita*	3	Bo, Ja	S, D
		*Ampelisca agassizi*	1	Bo	D
		*Ampelisca bicarinata*	2	Bz, Bo	S
		*Ampelisca schellenbergi*	1	Bo	S
		*Ampelisca* sp A	38	Bz, Bo, Ja	S, M, D
	Anamixidae	*Anamixis cavatura*	8	Bz, Bo, Ja	S, M, D
	Amphilochidae	*Hourstonius tortugae*	11	Bz, Bo, Ja	S, M, D
	Bateidae	*Batea cuspidata*	9	Bz, Bo, Ja	S, M, D
	Colomastigidae	*Colomastix janiceae*	14	Bz, Bo, Ja	S, M, D
	Cyprodeidae	Cyprodeidae A	2	Bo	S
	Dexaminidae	Dexaminidae A	1	Bo	S
		*Dexaminella* sp	5	Bz	D
	Iphimediidae	Iphimediidae A	6	Bz, Ja	S, D
	Leucothoidae	*Leucothoe laurensis*	3	Bz, Bo	D
		*Leucothoe spinicarpa*	102	Ja	S, M, D
		*Leucothoe* sp A	1	Bo	M
	Liljeborgidae	*Liljeborgia bousfieldi*	3	Bo	S
		*Liljeborgia* sp A	5	Bz, Bo, Ja	S, M, D
		*Listriella* sp A	2	Bz, Bo, Ja	S, D
	Lyssianassidae	*Concarnes concavus*	3	Bz, Bo, Ja	M
		*Hippomedon* sp A	1	Bz	M
		*Lyssianopsis alba*	22	Bz, Bo, Ja	S, M, D
	Megaluropidae	*Gibberosus myersi*	7	Bo, Ja	M, D
	Ochlesidae	*Curidia debrogania*	1	Ja	S
	Phoxocephalidae	*Eobrolgus spinosus*	24	Bz, Bo, Ja	S, M, D
		*Metarphinia floridana*	15	Bz, Bo, Ja	S, M, D
	Sebidae	*Seba tropica*	3	Bz	D
	Oedicerotidae	Oedicerotidae A	1	Bz	M
		*Periculodes cerasinus*	3	Ja	S, D
	Synopiidae	*Synopia ultramarina*	4	Bz, Ja	M, D
		*Metatyron triocellatus*	3	Bo	S
	Ingolfiellidae	*Ingolfiella* sp A	9	Bz, Bo, Ja	S, M, D
Isopoda	Gnathiidae	***Gnathia beethoveni***	3	Bz	S, D
		***Gnathia magdalensis***	53	Bz, Bo, Ja	S, M, D
		*Gnathia puertoricensis*	94	Bz, Bo, Ja	D
		***Gnathia vellosa***	64	Bz, Bo, Ja	D
		***Gnathia virginalis***	37	Bz, Bo	D
		*Gnathia* sp A	1	Ja	D
	Anthuridea	Anthuridea	3	Bz, Bo	S, M
	Anthuridae	***Amakusanthura magnifica***	73	Ja	S, M, D
		***Amakusanthura signata***	46	Bz, Bo, Ja	S, M, D
		Amakusanthura sp A	14	Bz, Bo	S, M
		***Anthomuda affinis***	1	Bo	M
		*Apanthura cracenta*	152	Bz, Bo, Ja	S, M, D
		***Apanthuroides millae***	1	Ja	S
		***Cortezura confixa***	2	Bz	S
		*Cyathura* sp A	1	Bo	S
		***Mesanthura bivittata***	5	Bz, Bo, Ja	S, M, D
		***Mesanthura hopkinsi***	8	Bz, Bo, Ja	D
		*Mesanthura paucidens*	8	Bz, Bo, Ja	S, M, D
		*Mesanthura pulchra*	24	Bo	S
		*Mesanthura* sp A	17	Bz, Bo, Ja	S, M
		***Pendanthura hendleri***	62	Bz, Bo, Ja	D
		*Pendanthura tanaiformis*	4	Bz, Bo	M
		*Pendanthura* sp A	2	Bz, Bo	S, D
	Expananthuridae	***Eisothistos petrensis***	2	Bz, Bo	D
		***Heptanthura scopulosa***	1	Ja	M
	Leptanthuridae	*Accalathura crenulata*	26	Bo, Ja	S; M
	Paranthuridae	***Colanthura tenuis***	3	Bz, Bo, Ja	S, M, D
		*Colanthura* sp A	3	Ja	S, D
		***Paranthura floridensis***	8	Bo, Ja	S, M
		*Paranthura infundibulata*	6	Ja	S, M
	Cirolanidae	*Anopsilana jonesi*	1	Bo	M
		***Calyptolana hancocki***	24	Bo, Ja	S, M, D
		*Cirolana albioida*	1	Bo	M
		***Cirolana crenulitelson***	1	Boc	M
		*Cirolana parva*	375	Bz, Bo, Ja	S, M, D
		*Eurydice convexa*	5	Bz, Bo, Ja	D
		*Neocirolana obtruncata*	3	Bon	M
		*Metacirolana agaricicola*	26	Bz, Bo, Ja	S, M, D
		***Metacirolana halia***	43	Bz, Bo	D
		***Metacirolana menziesi***	2	Bz, Bo	S, D
	Limnoriidae	*Limnoria platicauda*	3	Bz, Bo, Ja	S, M
	Corallanidae	*Alcirona krebsi*	2	Bo	M
		*Excorallana antillensis*	175	Bz, Bo, Ja	S, M, D
		*Excorallana berbicensis*	1	Bz	M
		*Excorallana tricornis*	6	Bz, Bo	S, M, D
		***Excorallana warmingii***	3	Bo	M, D
		*Excorallana* sp A	4	Bz, Ja	S, D
	Sphaeromatidae	*Cymodoce ruetzleri*	47	Bz, Bo, Ja	S, M, D
		***Dycerceis kensleyi***	1	Bz	M
		***Geocerceis barbarae***	108	Bz, Bo, Ja	S, M, D
		***Exosphaeroma diminuta***	2	Ja	S
		***Exosphaeroma yucatanum***	3	Ja	D
		*Exosphaeroma* sp A	9	Bz, Ja	S, D
		*Paracerceis caudata*	38	Ja	S, M, D
		*Paracerceis* sp A	1	Ja	S, D
	Janiridae	*Carpias algicola*	36	Bz, Bo, Ja	S, M, D
		*Carpias triton*	1	Bz	S
	Ganthostenetroidae	***Gnathostenetroides pugio***	37	Bz, Bo, Ja	S, M, D
		*Gnathostenetroides* sp A	4	Bz	S, D
	Joeropsisidae	***Joeropsis bifasciatus***	9	Bz, Bo, Ja	S, M, D
		***Joeropsis personatus***	4	Bo, Ja	D
		***Joeropsis rathbunae***	9	Bo, Ja	D
		***Joeropsis tobagoensis***	6	Bo, Ja	S, M
		*Joeropsis* sp A	15	Bz, Bo, Ja	S, M, D
	Stenetriidae	*Hansenium bowmani*	13	Ja	D
		***Hansenium stebbingi***	80	Bz, Bo, Ja	S, D
		***Hansenium spathulicarpus***	9	Bz, Bo, Ja	S, M, D
		***Stenetrium serratum***	13	Bz, Bo, Ja	D
		*Stenobermuda* sp A	1	Bo	S
		***Lyocoryphe minocule***	20	Ja	S, M, D
	Holognathidae	*Cleantioides planicauda*	1	Bz	M
	Idoteidae	*Erichsonella filiformis*	1	Ja	S
	Munnidae	***Uromunna reynoldsi***	4	Bz, Bo, Ja	S, M, D
	Paramunnidae	Paramunnidae A	1	Bz	D
	Pleurocopidae	***Pleurocope floridensis***	1	Bo	D
Tanaidacea	Apseudidae	Apseudidae A	2	Bo	S
		*Apseudes* sp A	488	Bz, Bo, Ja	S, M, D
		*Apseudes bermudeus*	58	Ja	D
		*Apseudes orghidani*	11	Ja	S, D
		*Hoplomachus propinquus*	5	Ja	S, D
	Kalliapseudidae	***Kalliapseudes bahamensis***	16	Ja	S, M, D
		***Psammokalliapseudes granulosus***	8	Bz, Bo, Ja	S, D
	Metapseudidae	*Apseudomorpha* sp A	179	Bz, Bo, Ja	S, M, D
		*Pseudoapseudomorpha* sp A	34	Bz, Bo, Ja	S, M
		*Synapseudes* sp A	132	Bz, Bo,	S, M, D
		*Discapseudes belizensis*	10	Bz	M
		*Parapseudes* sp A	16	Bz, Ja	S, D
	Pagurapseudidae	***Pagurotanais bouryi***	221	Bo, Ja	S, M, D
	Tanaididae	*Sinelobus stanfordi*	172	Bz, Bo, Ja	S, M, D
		*Zeuxo kurilensis*	51	Bz, Bo, Ja	S, M, D
	Leptocheliidae	*Hargeria rapax*	16	Bz, Bo,Ja	S, M
		*Leptochelia dubia*	834	Bz, Bo,Ja	S, M, D
		*Leptochelia longimana*	4	Bz, Bo, Ja	S
		*Pseudoleptochelia* sp	1,164	Bz, Bo, Ja	S, M, D
	Nototanaidae	Nototanaidae A	26	Bz, Bo, Ja	S, M, D
		Nototanaidae C	62	Bz, Bo, Ja	S, M, D
	Paratanaidae	*Paratanais* sp A	1,433	Bz, Bo, Ja	S, M, D
Cumacea	Bodotriidae	*Vaunthompsonia floridana*	4	Bz	S, M, D
		*Vaunthompsonia minor*	4	Bz, Bo	S, D
		*Vaunthompsonia* sp A	1	Bz	D
		*Mancocuma* sp A	6	Bz, Bo, Ja	M, D
		*Spilocuma* sp A	1	Ja	D
		*Cyclaspis goesi*	14	Bz, Bo, Ja	S, M, D
		***Cyclaspis granulata***	1	Bo, Ja	D
		**Cyclaspis varians**	1	Bo, Ja	S, M
		*Cyclaspis* sp A	6	Bo, Ja	D
	Leuconidae	*Eudorella* sp A	3	Bz	D
		*Leucon* sp A	3	Bz, Bo, Ja	S, D
		*Leucon* sp B	1	Bz	D
	Nannastacidae	*Campylaspis heardi*	3	Bz, Ja	S, M, D
		*Campylaspis* sp A	3	Bz, Bo	S, D
		***Cumella antipai***	4	Bo, Ja	S
		*Cumella clavicauda*	11	Bz, Bo, Ja	S, M, D
		*Cumella garrityi*	6	Ja	D
		***Cumella gomoiui***	5	Bz, Bo, Ja	S, M, D
		*Cumella longicaudata*	7	Bz, Bo, Ja	S, M, D
		***Cumella meredithi***	1	Bo	D
		***Cumella murariui***	6	Bz, Bo, Ja	S, M, D
		*Cumella ocellata*	18	Bz, Bo, Ja	S, M, D
		***Cumella ruetzleri***	7	Bz, Bo, Ja	S, M, D
		***Cumella serrata***	15	Bz, Bo, Ja	S, M, D
		*Cumella vicina*	22	Bz, Bo	D
		Cumella sp A	1	Bo	S
		Cumella sp G	8	Bz, Bo, Ja	S, M, D
		***Cubanocuma gutzi***	9	Bz, Bo, Ja	D
		*Elassocumella* sp A	2	Ja	S, M
		***Schizotrema aglutinanta***	7	Bz, Bo, Ja	S, M, D

**Figure 2:**
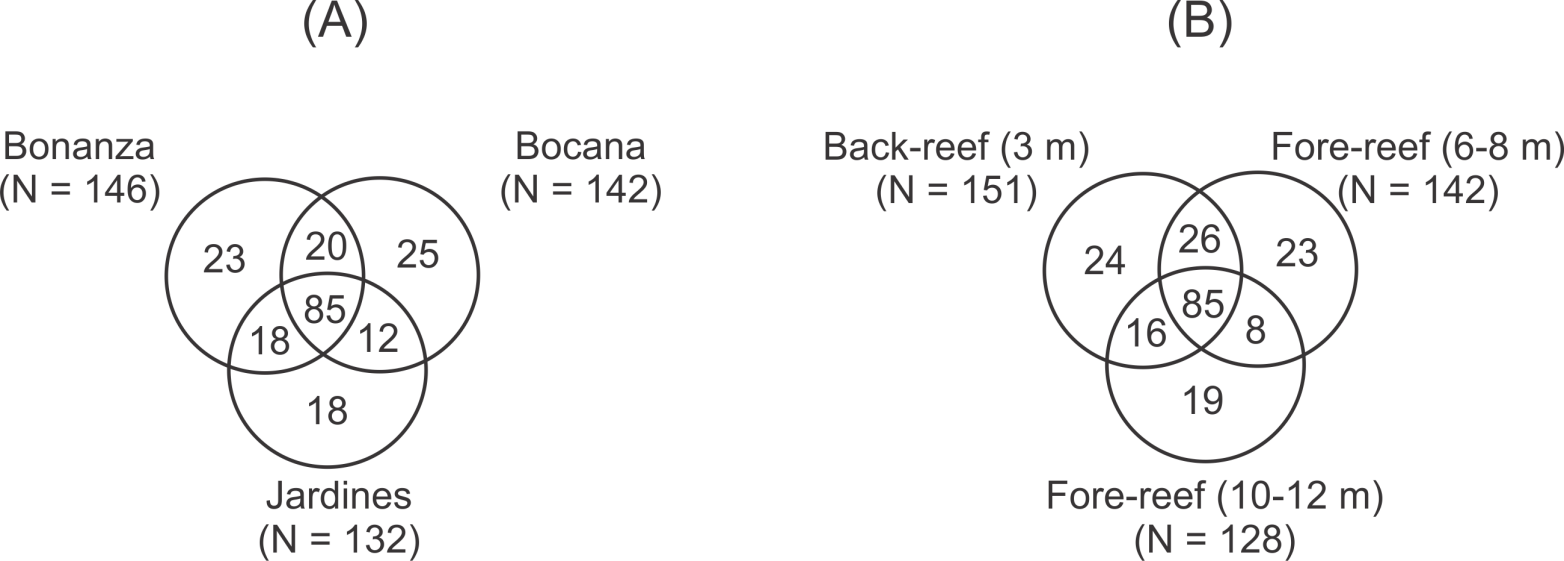
Venn diagrams illustrating the unique and shared taxa of peracarid crustaceans among (A) reef sites and (B) depth within the Puerto Morelos Reef National Park, Mexico.

The number of peracarid taxa didn’t differ significantly (2-way ANOVA, *p* > 0.05) among sites (Bonanza = 146, Bocana = 142, Jardines N = 132) and depths (shallow = 151, medium = 142, deep = 128), but the composition of the assemblage was heterogeneous and only 20 taxa were shared among all sites and depths. Venn diagrams illustrate that 85 of the 200 taxa recorded were shared by the three reef sites (43%; Fig. 2A), with Bocana having the highest number of unique taxa (*N* = 25). The three depths also shared 85 taxa, with the shallow back-reef having the highest number of unique taxa (*N* = 24) (Fig. 2B).

**Figure 3:**
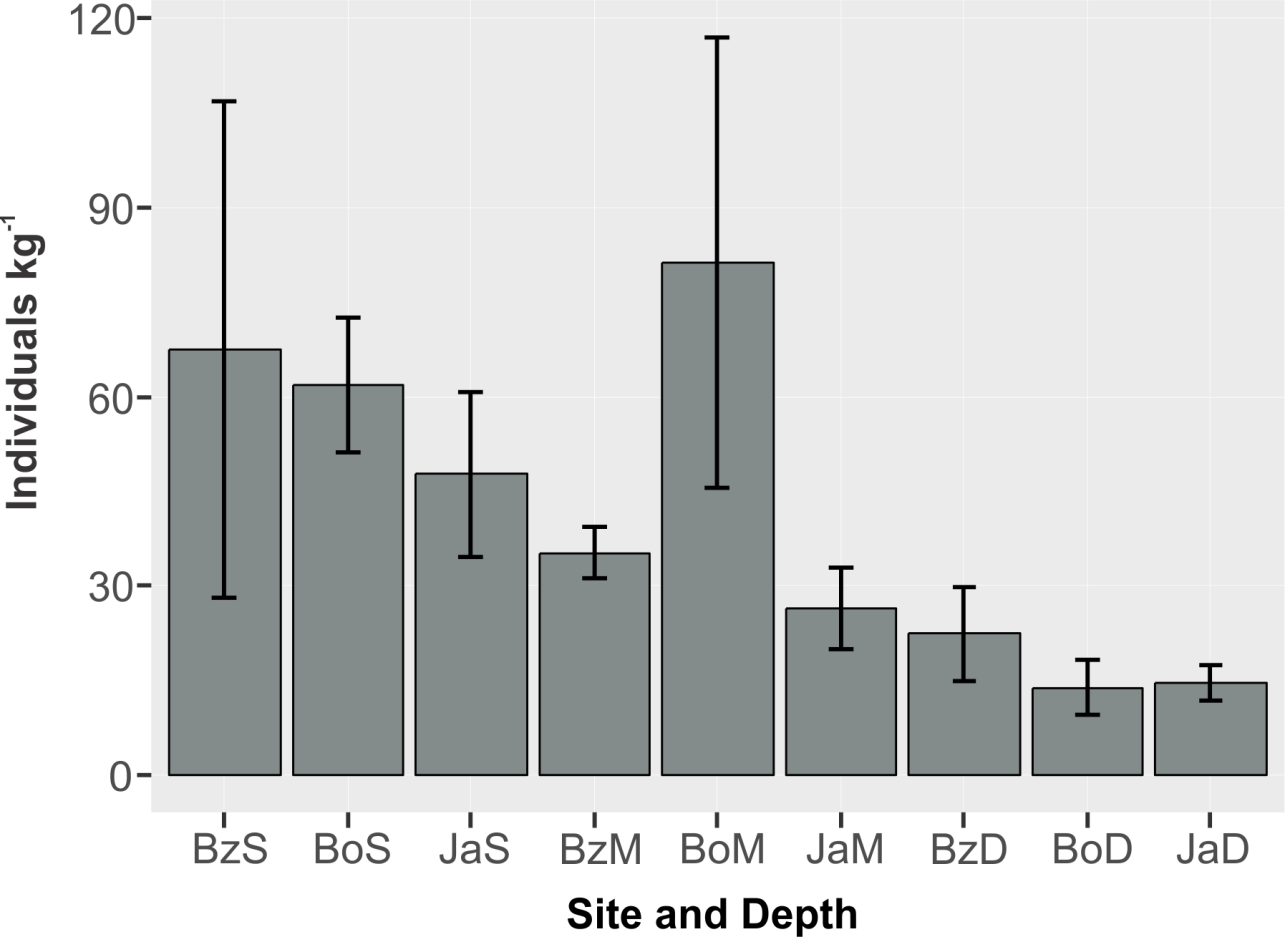
Abundance (individuals kg^−1^) (mean and standard error) of cryptic peracarids in coral rubble in three depths (S = shallow back-reef (3 m), M = intermediate depth fore-reef (6–8 m), D = deep fore-reef (10–12 m) of three reef sites (Bz: Bonanza, Bo = Bocana, Ja = Jardines) within the Puerto Morelos Reef National Park.

The mean abundance of peracarids (Fig. 3) was not significantly different between reef sites (2-way ANOVA, *p* > 0.05; Table 2), while differences were significant between depth zones (*p* < 0.01), with abundance being significantly lower (TukeyHSD, *p* < 0.01) in the deep (10–12 m) fore-reef (Mean = 17.1, SE = 3.0 individuals kg^−1^) than in the shallow (3 m) back-reef (Mean = 59.0, SE = 13.1 individuals kg^−1^).

**Table 2:** Results of the 2-way ANOVA of the abundance of peracarids in coral rubble. Fixed factors were site (Bonanza, Bocana, Jardines) and depth (shallow back-reef (3 m), intermediate depth fore-reef (6–8 m) and deep fore-reef (10–12m)).

Source	*df*	SS	MS	*F*	*p*
Site (S)	2	0.1582	0.0790	0.703	0.5038
Depth (D)	2	1.5819	0.7907	7.031	0.0035^**^
S × D	4	0.2472	0.0618	0.549	0.7010
Residual	27	3.0371	0.1125		

**Notes.**

** indicates a significant difference (*p* < 0.05).

**Figure 4:**
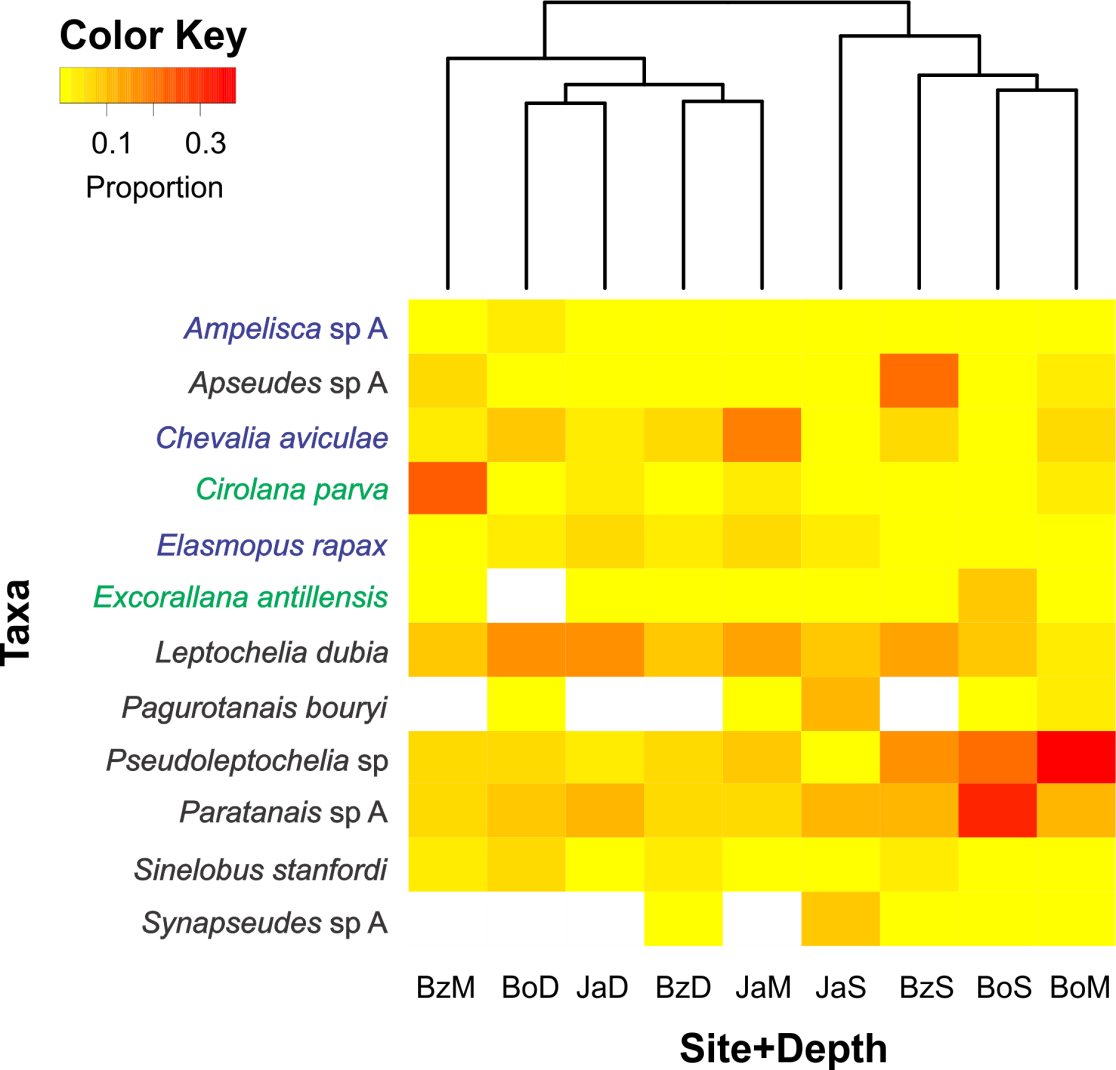
Relative abundance heatmap of cryptic peracarid taxa encountered within each site (Bz: Bonanza, Bo: Bocana, Ja: Jardines) and depth (S: shallow back-reef, M: fore-reef (6–8 m), D: fore-reef (10–12 m)) surveyed within the Puerto Morelos Reef National Park in 2013–2014. A Bray–Curtis dissimilarity dendogram on the top highlights the taxonomic dissimilarity among sites and depths. The 200 peracarid taxa were used for the clustering but only those that had a relative abundance higher than 5% are shown in the heatmap. Colour scale shows the proportion of each taxa within each site and depth. White squares indicate zero counts. Taxa in blue correspond to amphipods, in green to isopods and in black to tanaidaceans.

Of the 200 taxa collected, only 12 had relative abundances higher than 5% (Fig. 4). Dominant taxa in most sites and depths were the tube-dweller tanaidaceans *Pseudoleptochelia* sp A, *Paratanais* sp A, and *Leptochelia dubia*. Other abundant taxa were *Apseudes* sp A, in the shallow site of Bonanza, *Cirolana parva*, in the medium depth of Bonanza, and *Chevalia aviculae*, in the medium depth of Jardines (Fig. 4). Hierarchic clustering of the abundance of all 200 taxa revealed two clusters, one formed by the three deep sites and the medium depth site of Bonanza, and another formed by the shallow sites and the medium depth sites of Bocana and Jardines (Fig. 4).

## Discussion

The occurrence of 200 taxa of cryptic peracarids within the PMRNP shows a high taxonomic richness and highlights coral rubble as an important biotope for this superorder. Furthermore, the identification of 50 new records of peracarid species for the Mexican Caribbean Sea contributes to reduce regional gaps in the knowledge of this superorder. Taxonomic richness of Isopoda (*N* = 75), Tanaidacea (*N* = 22) and Cumacea (*N* = 30) recorded for the PMRNP is higher than reported for other coastal habitats in the Mexican Caribbean, and in the case of Tanaidacea it was higher than previously reported for the Caribbean Sea (Table 3). By contrast, the number of taxa of Amphipoda recorded in the PMRNP (*N* = 72) is lower and accounts for approximately one fourth of that previously reported for the Mexican Caribbean Sea and one tenth for the Caribbean Sea (Table 3). The mysid fauna recorded in the PMRNP was low (Taxa = 1), similar to previous reports for the Caribbean, however, it should be noted that coral rubble is not the preferred habitat of Mysidacea, as most of the known species (>1,000) are free living and inhabit coastal and open sea waters (Meland, 2002 onwards). Further reef studies that include different habitats, zones and depths, and that employ different methods (i.e., benthic cores, nets, light-traps) in order to sample different components or guilds (Costello et al., 2017), will probably yield a much higher taxonomic richness of peracarids for the Mexican Caribbean.

**Table 3:** Taxonomic richness of peracarid fauna recorded in this study, in the Mexican Caribbean and in the Caribbean Sea.

Order	Caribbean sea	Mexican Caribbean sea	This study
Mysida	5^a^	4^b^	1
Amphipoda	535^c^	266^d^	72
Isopoda	178^e^	51^f^	75
Tanaidacea	19^g^	20^h^	22
Cumacea	35^i^	15^j^	30
Total	772	356	200

**Notes.**

a Sorbe, Martin & Diaz (2007).

b Markham & Donath-Hernández (1990).

c Martín et al. (2013).

d Martín et al. (2013).

e Kensley & Schotte (1989).

f Markham et al. (1990), Cantú-Díaz Barriga & Escobar-Briones (1992), Van Tussenbroek, Monroy-Velazquez & Solis-Weiss (2012).

g Ortiz & Lalana (1993).

h Suárez-Morales et al. (2004).

i Petrescu (2002).

j Donath-Hernández (1988).

In addition to the high taxonomic richness, our study shows that the number of taxa and the abundance of cryptic peracarids were higher in shallow back-reef areas and decreased with depth. Similar patterns of decreased abundance of peracarids with depth have been reported by Campos-Vásquez et al. (1999) and López et al. (2008), who proposed that cryptofauna assemblages are affected by interstitial sediment as a limiting factor, and by variations in flushing. We propose that the higher taxonomic richness and abundance of cryptic peracarids in the shallow back-reef of the PMRNP are related to reef development and to the amount of coral rubble produced during hurricanes. Puerto Morelos reef is best developed in back-reef and reef-crest zones, where it is dominated by the branching coral *Acropora palmata*, and has a poorly developed fore-reef zone that lacks the spur and groove systems that characterize other Caribbean reefs and has low scleractinian coral cover (Jordán, 1979; Jordán-Dahlgren, 1989). In the late 1970’s, coral cover on this reef was 43% in the back-reef, 33% in the reef crest and 7% in the fore-reef (Jordán, 1979). In 1988, the reef was impacted by hurricane Gilbert (category V in the Saffir-Simpson Scale) which caused a reduction in scleractinian coral cover by 89% in the back-reef zone, by 81% in the reef-crest and by 68% in the fore-reef zone (Rodríguez-Martínez, 1993). After hurricane Gilbert, Puerto Morelos reef was impacted by hurricanes Roxane (category III—1995), Ivan (category V—2004) and Wilma (category V—2005), increasing the accumulation of coral rubble in the back-reef zone even further and creating adequate habitat for cryptic species. Dead coral fragments and coral rubble provide a better habitat for cryptic species than living corals, which can display several defense mechanisms (Lang & Chornesky, 1990), and through the creation of microhabitats, which favor diversity (Takada, Abe & Shibuno, 2007; Takada et al., 2016; Enochs, 2012). Coral rubble is also a favorable substrate for the growth of algal turf which provides food, substrate and protection for cryptic peracarids (Klumpp, McKinnon & Mundy, 1988).

No significant differences in taxonomic richness and abundance of cryptic peracarids were observed between reef sites suggesting that, at the time of the surveys, environmental conditions were similar throughout the PMRNP for this superorder. Nevertheless, the closeness of Puerto Morelos coral reef (<3.5 km) to a coast that is experiencing intensive land development, as a result of the rapid growing tourism industry (Metcalfe et al., 2011), and where there is inadequate treatment of waste waters (Rodríguez-Martínez et al., 2010), could affect the health of the coral reef in a short-time frame (<10 years). Benthic crustacean communities are good bioindicators of water quality and reef health, as they are sensitive to changes in environmental variables (Grahame & Hanna, 1989; Conradi, 1995) and have been shown to respond to perturbation either by reducing or by increasing their abundance (Snelgrove & Lewis, 1989; Chintiroglou et al., 2003; De-la-Ossa-Carretero et al., 2010; Enochs et al., 2011). Peracarid data obtained in the present study can be used as a baseline for future monitoring programs in the PMRNP. Monitoring the abundance of these taxa, and their relation with physicochemical parameters, could help detect changes in water quality (Esquete, Moreira & Troncoso, 2011). Monitoring could also help to recognize invasive species (Costello et al., 2017). In the present study we recorded two species, *Ampelisca abdita* and *A. schellenbergi*, reported as invasive by Winfield et al. (2011), who suggested that they probably arrived in the Gulf of Mexico in ballast water, which is not regulated in Mexico (Okolodkov et al., 2007). Invasive peracarids could also arrive to the PMRNP through floating weeds. In 2014–2015, the Mexican Caribbean coastline received massive arrival of pelagic *Sargassum* that reached peak values of 19,603 m^3^ km^−1^ in September 2015 (Rodríguez-Martínez, Van Tussenbroek & Jordán-Dahlgren, 2016). The cause of this atypical event was unknown, and it remains to be seen if it will become cyclical, in which case peracarid taxonomic richness and abundance could change rapidly.

## Conclusions

Cryptic peracarid crustaceans in coral rubble are diverse and abundant within the PMRNP. Taxa richness of the orders Isopoda, Tanaidacea and Cumacea was larger than previously reported for coral rubble and other costal habitat types in the Mexican Caribbean, while that of Amphipoda was lower. The most abundant order was Tanaidacea with dominant species belonging to the families Paratanaidae and Leptocheliidae. Within the reef system taxonomic richness and abundance of cryptic peracarids were higher in the shallow back-reef areas than in the reef front, where values decreased with depth. This elevated occurrence in the back-reef may result from a larger accumulation of coral rubble, which occurs during hurricanes. No significant differences in taxonomic richness and abundance of cryptic peracarids were observed between reef sites suggesting homogeneous environmental conditions for this superorder across the PMRNP. The data obtained in the present study can serve as a baseline for future monitoring programs in the PMRNP that aim to detect changes in water quality and invasive species.

##  Supplemental Information

10.7717/peerj.3411/supp-1 Supplemental Information 1

10.7717/peerj.3411/supp-2 Supplemental Information 2 DataHM

10.7717/peerj.3411/supp-3 Supplemental Information 3 Pecarids heatmap

10.7717/peerj.3411/supp-4 Supplemental Information 4 Raw data

10.7717/peerj.3411/supp-5 Supplemental Information 5 R code

## Additional Information and Declarations

### Field Study Permissions

The following information was supplied relating to field study approvals (i.e., approving body and any reference numbers): SAGARPA (Agriculture, Natural Resources and Fisheries Secretariat, Mexico).

### Data Availability

The following information was supplied regarding data availability: GitHub: https://github.com/rerodriguezmtz/Peracarids.
